# Effect of Color Light Stimulation Using LED on Sleep Induction Time

**DOI:** 10.1155/2017/6030268

**Published:** 2017-05-11

**Authors:** Seonjin Lee, Dongwook Kim

**Affiliations:** ^1^Department of Healthcare Engineering, Graduate School, Chonbuk National University, Jeonju, Republic of Korea; ^2^Department of Biomedical Engineering, Chonbuk National University, Jeonju, Republic of Korea; ^3^Research Center of Healthcare and Welfare Instrument for Aged, Chonbuk National University, Jeonju, Republic of Korea

## Abstract

The effects of color are already being used widely. For this reason, in this study, an attempt was made to use such effects of color to examine the changes in sleep onset through the use of the preferred and nonpreferred color light stimulation. Color light stimulations were randomly presented to the subjects, and based on these colors, the changes in sleep onset were examined through the EEG. Also, to quantify the physiological changes that were caused by each color light stimulation, the changes in the HRV were examined through ECG to determine the level of activation of the autonomous nervous system. The results showed that sleep onset time was changed based on the light stimulation. The result of the EEG analysis showed that sleep onset time was most significantly shortened in preferred color light stimulation. Also, the result of HRV was the fastest change about both the time domain and the frequency domain in the preferred color light stimulation. Therefore, because the preferred color light stimulation activated the parasympathetic nervous system, sleep was induced quickly. Also, by simply using the HRV, the differences in the index of HRV showed changes of sleep onset according to the color light stimulation.

## 1. Introduction

In the modern society, the contact with external areas has become more frequent and complicated, and due to the diversified technical development, the lives of human beings have become richer, ascending to a higher quality level. The people making up the modern society, however, who are exposed to a stream of new information, have to suffer from stresses, and due to the technological progress, the time that they could devote to activities has increased, shortening the time available for sleep. This reduced the amount of stress that could be relieved through sleeping. Stress is known to take its toll not only on the physical health but also on the mental health of people.

Stress is a state of failure to adapt to the various stimulations or changes that occur in one's life. Also, stress may overactivate the sympathetic nervous system in an acute or chronic way, causing psychological and social problems [1]. The factors that invoke stress include environmental and mental factors, and they work in conjunction with one another. A proper level of stress may be a source of motivation in people's daily lives, but excessive stress may cause various diseases. Medical studies have also shown that stress hinders people's routine activities as well [2].

There are many diseases that are affected by or caused by stress. Stress affects the gastric, respiratory, and endo-secretion systems as well as all the other internal systems of the human body. Also, it can cause various psychological disorders, such as depression, difficulty in focusing, and sleep deprivation. According to ICSD's (International Classification of Sleep Disorders) diagnostic standard on sleep deprivation, primary insomnia is fundamentally caused by stress [3]. Particularly, it was found in a study on the adults in South Korea that about 20% of them were suffering from insomnia [4]. In addition, according to the study, 31% of the adult population in South Korea experience difficulty in sleeping, such as abrupt interruption or termination of sleep, depriving such people of a full rest [5]. Therefore, it appears that the people making up the modern society are suffering from sleep disorders.

Insomnia is a disorder in which the patient finds it difficult to fall asleep and to maintain the sleeping state, or whose sleep is often terminated, depriving him or her of a full rest [6]. According to DSM-IV (Diagnostic and Statistical Manual of Mental Disorders, 4th edition), an American manual for classifying mental diseases, insomnia may occur in an acute form when the patient is exposed to psychological, social, or medical stress [7]. In fact, 78% of insomnia patients report that the onset of the disease was affected by stress [7].

Of the people diagnosed with insomnia, 5~36% visit clinics with insomnia as the main symptom, but most of them were diagnosed with insomnia while being treated for other diseases at the clinic [8]. In reality, however, insomnia patients tend to evade treatment of sleep induction even though they understand its necessity due to their groundless worries about sleep induction and about the side effects of drugs like sleeping pills.

In this study, for this reason, colors, which are commonly used in people's daily lives, were used to investigate sleep induction, without using drugs, to minimize the anxiety or worries of the patients. Preferred and nonpreferred color light stimulations were provided through LED devices, and the effects of such stimulation in terms of sleep induction were analyzed by measuring the subjects' electroencephalography (EEG), which are physiological signals. Also, the impact of the color light stimulation on the human body was quantified through the heart rate variability (HRV). With this, the asleep and awake states were compared in terms of their physiological indices. An attempt was also made to detect sleep more easily using the HRV analysis.

## 2. Equipment and Methods

In this study, the subjects' EEG and HRV were measured to determine the physiological impact of color light stimulation and the changes that occur in terms of sleep induction depending on the color light stimulation (preferred and nonpreferred colors).

### 2.1. Electroencephalogram Recording Equipment

The EEG is a noninvasive method using the electric potential resulting from the synthesis of the electric activities on the neurons [9]. It has spatial, chronological, and frequency-based characteristics, which are related to the functioning of the human brain [10]. Normally, based on the frequency domain, the EEG is classified into delta (0.5~4 Hz), theta (4~8 Hz), alpha (8~13 Hz), beta (13~30 Hz), and gamma (30~50 Hz) waves [11].

The frequency bands of brain waves that occur during sleep differ depending on the stage of sleep. As such, these are used as indices in sleep-related studies [12]. Sleep consists of the rapid eye movement (REM) stage, which involves rapid eyeball movements, and the nonrapid eye movement (NREM) stage, without such movements. These two sleep stages are iterated at regular intervals. In the NREM stage, there are five different stages of sleep: awake and asleep stages 1 to 4.

In this study, the EEG measurement was taken using a Vision Recorder (Brain Products GmbH, Germany). The electrodes were located at the F4-A1, C3-A1, C4-A1, O1-A1, and O2-A1 areas, as recommended by AASM (American Academy of Sleep Medicine). When the EEG measurement was taken, the ground electrode was attached to the right ear, and in accordance with the international 10-20 system, a total of seven electrodes were attached.

### 2.2. Heart Rate Variability Recording Equipment

During sleep, the parasympathetic nervous system is activated, and as in the previous study, the different stages of sleep show significant differences in terms of the activities of the autonomic nervous system (ANS) [13]. A change occurs in the ANS according to sleep progresses. The parasympathetic nervous system is mainly activated during NREM-sleep than when it is relaxing with awakening. On the contrary to this, in the case of rapid eye movement REM-sleep, sympathetic nervous system activity was increased. Therefore, when NREM-sleep occurs, the heart rate, cardiac output and blood pressure, and so forth are lower than awake state. However, heart rate and blood pressure rise irregularly during REM-sleep [14]. The HRV, which uses the R-R interval (RRI) of electrocardiogram (ECG), is used to evaluate the ANS. The HRV is the quantified value of the variations in heart rate, which continuously changes between the heart rate intervals and is used as a noninvasive method of showing the changes that occurred in terms of the ANS [15, 16]. Therefore, the HRV responds to the level of sleep and awake stage of the subject with a level of sensitivity, making it also popular among sleep-related studies [17]. The HRV can be analyzed in the time and frequency domains. First, the time domain consists of the mean R-R, which shows the average of interval between the R peaks; the SDNN, which is the standard deviation of the intervals of all RRIs; rMSSD, the standard deviation of the average square roots of the differences in the RRIs; and pNN50, which is the relative ratio of the number of NN50 that indicating the difference between nearby RRIs exceeding over 50 ms [18]. Because the parasympathetic nerve is activated when the subject is sleep on 1 stage of sleep, the heart rate is decreased then the SDNN, RMSSD, and pNN50 tend to increase, while they tend to decrease when the subject is awaken. The frequency domain analysis is used to classify the ECG frequencies in terms of the bands. Therefore, the domains consist of the very low-frequency power (VLF), which shows the hormone effects based on the relative power, the low-frequency power (LF), which is the activation level of the sympathetic nervous system; and the high-frequency power (HF), which shows the activation level of the parasympathetic nervous system and is a vagal tone maker [19, 20]. Also, there is the low-frequency power to that high-frequency power ratio (LF/HF ratio), which indicates the relative balance in the activation of the parasympathetic and sympathetic nervous systems [19]. The LF/HF ratio represents an index of sympatho-vagal balance, with higher values indicating increased relative sympathetic dominance [20]. In the previous studies on the LF/HF ratio, the average of a healthy adult was found to be 1.7 [21]. A lower LF/HF ratio means that the activities of the parasympathetic nervous system increase while those of the sympathetic nervous system decrease [15]. This reflects that the balance of the ANS was lopsided toward the parasympathetic nerve. As such, the HRV is used as an important tool for determining the quality of sleep. For this reason, it could be used as indices for dividing the awake and asleep states. In this experiment, MP150 (Biopac System Inc., USA) was used to record the ECG.

### 2.3. Color Light Simulation Equipment

**Figure 1:**
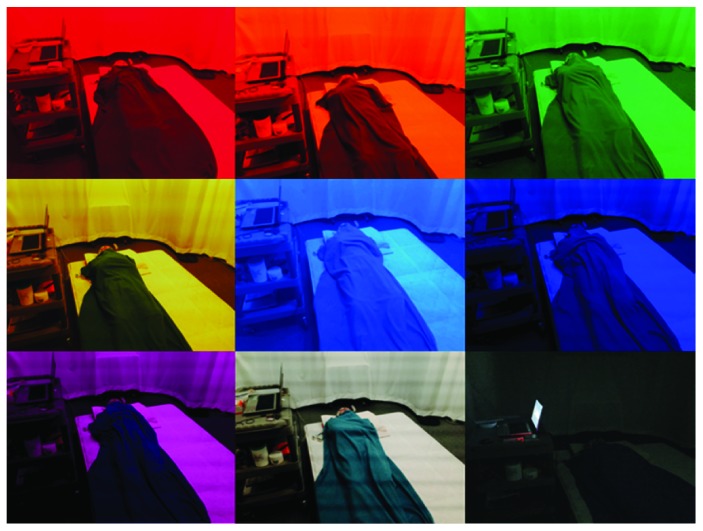
Example of color light stimulation experiments.

In this study, the physical signals were measured in a soundproof dark room to exclude external stimulation. The lighting stimulator that was used was direct lighting, using LED devices directly mounted on the ceiling. The color light stimulation were red, orange, yellow, green, blue, navy, and violet (Figure 1). In order to describe a general sleep environment, we set the “dark state” that turned on the support lightning lamp which helps the sleep. The brightness of dark state was 15 lux. The white light stimulation and dark state were used in the control group. The indoor temperature of the soundproof, dark room was 25 ± 1°C, and the humidity was 50 ± 5%.

### 2.4. Participants

10 adult male subjects were enrolled in this study (Table 1). None of them had any color or vision impairment. The subjects with mental disease histories or brain-related diseases were excluded from the study. Also, the subjects were instructed to sleep sufficiently before participating in the experiment, and the subjects who were on medications that could affect the central nervous system no later than 6 months before the test were excluded from the study. During the experiment period, the subjects were controlled to maintain their normal sleeping intervals. Table 1 shows subjects' characteristics. This study was approved by the Institutional Review Board (IRB) of Chonbuk National University (IRB File no.: JBNU 2015-12-005-001).

### 2.5. Experimental Procedure

**Figure 2:**
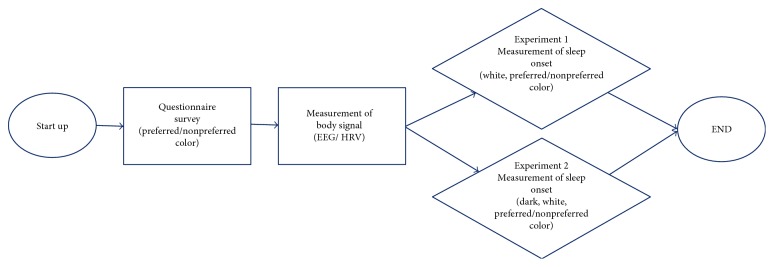
Protocol of experiments.

The experiments consisted of two. The subjects were interviewed before the experiment for the selection of a preferred color and a nonpreferred color, respectively. To investigate the change of sleep induction time in the bright state and the general state, it was classified into experiment 1 and experiment 2, respectively. In experiment 1, the white light stimulation and the selected preferred and nonpreferred color light stimulation were presented randomly to the subjects. In experiment 2, the white light, the selected nonpreferred color light, and the preferred color light stimulation were presented to the subjects, along with an additional dark state which was nonstimulation environment without any color light stimulation. Therefore, the subjects who participate in experiment 2 encountered a total of four situations: nonstimulation and color light stimulation (white, preferred color, and nonpreferred color). In case of experiment 1, white light stimulation was set as control, and in case of experiment 2, white light stimulation and dark were set as control. In all the tests, EEG and HRV were used to determine the effect of the colored lights, and the state of sleep was determined (Figure 2).

### 2.6. Analysis

In this study, 2 hours of experiment were conducted and for the measurement data for analysis, EEG and ECG data of 30 minutes in total were used, using 15 minutes before and after the segment where sleep was started. EEG analysis was performed for determining the subject's state of sleep, using BESA 5.1 (BESA GmbH, Germany). To remove the noise and artifacts, the bandpass and notch filters were used, and only the 0.5~60 Hz frequency band were extracted for the analysis. Also, through fast Fourier transform (FFT) analysis, each frequency band was divided into delta (0.5~4 Hz), theta (4~8 Hz), alpha (8~13 Hz), beta (13~30 Hz), and gamma (30~50 Hz) waves. To determine the state of sleep in this study, the EEG was divided into epoch (30 seconds) units: awake stage and asleep stage 1. If the relative power of the alpha wave was less than 50% and the relative power of the seta wave was higher than 23%, this is sleep stage 1 [22]. Therefore, in this study, the frequency properties of the EEG were used to classify the state in awake stage or asleep stage 1, and then the sleep induction time was extracted. In addition to quantify the physiological changes that occurred due to the color light stimulation, the HRV, through the RRI of the ECG, was used to measure the activities of the ANS. In this experiment, the gathered ECG data were processed using AcqKnowledge 4.2 (Biopac System Inc., USA) to extract the RRIs. Also, the obtained HRV values were separately analyzed for the time and frequency domains.

As the index of the state of sleep in the time domain analysis of the HRV, the mean R-R, SDNN, rMSSD, and pMM50 were used. Also, among the frequency domain analysis indices, the LF/HF ratio was only used. In this study, since measurement was conducted up to 1 stage of sleep, it became NREM-sleep, and the activity of parasympathetic nervous system was promoted during this period. When the parasympathetic nerve is activated, since the heart rate is decreased, HRV variations are changed. After the sleep was started, the indices of time domain (mean R-R, SDNN, rMSSD, and pNN50) were rapidly increased, while the LF/HF ratio was decreased. Therefore, in this study, the segment where the indices suddenly changed was set as sleep onset time.

To determine the relationships between the color light stimulation and sleep, SPSS 18.0 was used for statistical verification, which was based on the *p* = 0.05 significance level. The paired *t*-test method was also used to compare the changes between the awake and asleep states and the sleep induction time for each color light stimulation.

## 3. Results

**Figure 3:**
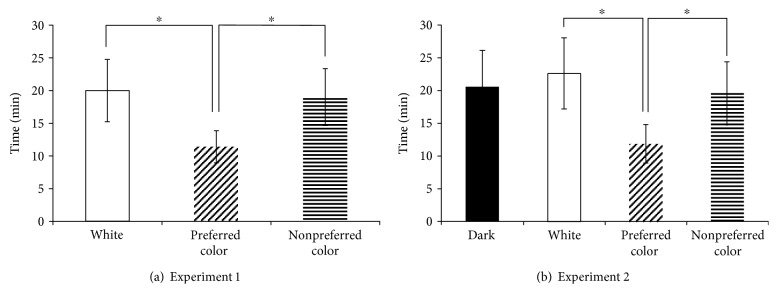
Time of sleep onset according to EEG analysis (^∗^ *p* < 0.05; each stimulus).

Based on the color light stimulation presented, the experiments were divided into experiment 1 and experiment 2. In the EEG result of experiment 1, the sleep onset was occurred at 18.8 min with the white light stimulation, at 11.8 min with the preferred color light stimulation, and at 18.1 min with the nonpreferred color light stimulation (Figure 3(a)). In experiment 2, the sleep onset was occurred at 20.4 min in the dark state, at 21.2 min in white light stimulation, at 12.3 min in the preferred color light stimulation, and at 17.0 min in the nonpreferred color light stimulation (Figure 3(b)). With experiment 1, the preferred color light stimulation significantly shortened the time of sleep onset compared to the white light and nonpreferred color light stimulation (*p* < 0.05). In experiment 2, the time of sleep onset was significantly shortened with the preferred color light stimulation compared to the nonpreferred color light stimulation and the dark state (*p* < 0.05). Also, compared to the dark state, the preferred color light stimulation had a tendency to shorten the time it took to induce sleep.

In experiment 1 and experiment 2, respectively, the changes of time and frequency domain index of HRV was presented according to awake-asleep states (Table 2). In both experiment 1 and experiment 2, the mean R-R, SDNN, rMSSD, and pNN50, which were the time domain indices, all increased while the subject was asleep rather than awake. The LF/HF ratio decreased during the subject was asleep.

**Figure 4:**
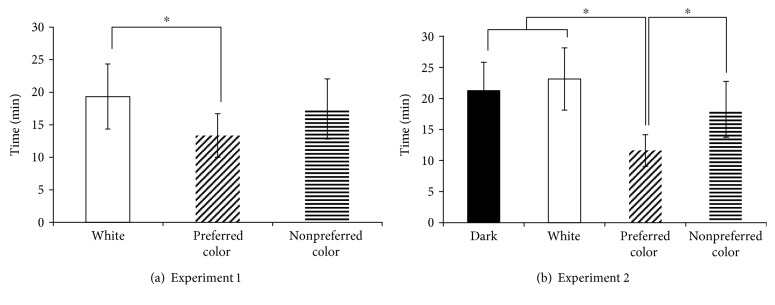
Time of sleep onset according to HRV analysis (^∗^ *p* < 0.05; each stimulus).

In addition, using the differences in terms of the time and frequency domain indices of the HRV based on the awake-asleep states, the changes that occurred in the sleep onset time due to the presentation of the color light stimulation were shown through the HRV, in a simplified method. In the case of the result where the HRV was used to indicate the time of sleep onset (experiment 1), the sleep onset was occurred at 19.3 min in white light stimulation, at 13.3 min in the preferred color light stimulation, and at 17.5 min in the nonpreferred color light stimulation (Figure 4(a)). In the results of experiment 2, the time of sleep onset at 21.1 min in dark state, at 21.2 min in the white light stimulation, at 12.3 min in the preferred color light stimulation, and at 16.8 min in the nonpreferred color light stimulation (Figure 4(b)).

In the case of experiment 1, the time of sleep onset was significantly shortened in the preferred color light stimulation compared to the white light stimulation (*p* < 0.05). Also, it had a tendency that the preferred color light stimulation to shorten the time of sleep onset compared to the nonpreferred color light stimulation. In the case of experiment 2, preferred color light stimulation was significantly the most shortened the time of sleep onset compared to the white light stimulation, dark state, and nonpreferred color light stimulation (*p* < 0.05).

**Figure 5:**
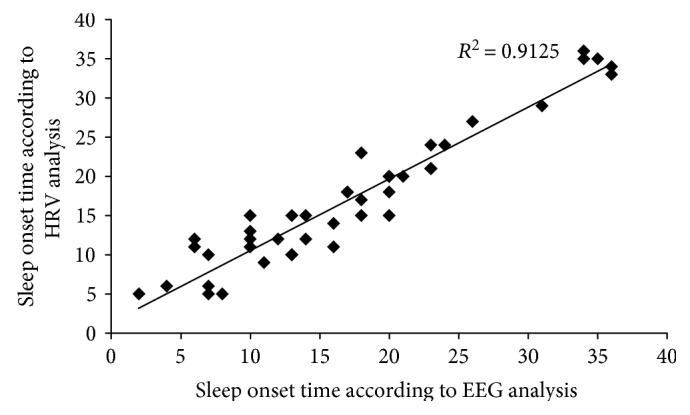
Correlationship of sleep onset results between EEG and HRV analysis.

To determine the correlations between the sleep onset times identified through the EEG and HRV, Pearson's bivariate analysis was conducted. The result showed a positive correlation, with *r* = 0.955 and *q* = 0.000 (Figure 5).

## 4. Discussion

In this study, the changes that occurred in the sleep onset times based in the preferred and nonpreferred color light stimulations were investigated through the EEG. And to show the effect of the color light stimulation on human physiology, the EEG was measured concomitantly with the HRV. Furthermore, an attempt was made to detect sleep more easily using the HRV analysis.

In the time of sleep onset results that were obtained using EEG, in both experiment 1 and experiment 2, the preferred color light stimulation showed the fastest sleep onset results.

Based on the result of the HRV using the ECG, the time domain indices increased significantly in the asleep state compared to the awake state. The LF/HF ratio, an index for the frequency domain, decreased under the asleep state. This is because in the state of NREM-sleep, the parasympathetic nervous system is activated. Therefore, the indices of time domain (Mean R-R, SDNN, rMSSD, and pNN50) were rapidly increased, while the LF/HF ratio was decreased. In this study, the segment where the indices suddenly changed was set as sleep onset time.

The HRV results for each color light stimulations showed that the environment of preferred color light stimulation, where sleep was induced the earliest, showed the highest levels of the time domain indices. Moreover, in the frequency domain, the LF/HF ratio was the lowest in this case. On the other hand, the environment of white light stimulation, where sleep was also the latest to occur, the time domain indices tended to be lower, and the LF/HF ratio was relatively higher. This means that the preferred color light stimulation activated the parasympathetic nervous system. For this reason, it can be said that the color light stimulation induced a state of stabilization.

Lastly, the detection of the sleep onset time through a simplified method utilizing the differences between the HRV indices also showed a difference in the average changes that occurred with the time of sleep onset. In experiment 1, the HRV indices showed faster time of sleep onset under the preferred color light stimulation compared to the others (white light, nonpreferred color light stimulation). For this reason, it is believed that the preferred color light stimulation resulted in a faster achievement of the asleep state. Also, in the case of experiment 2, the white light and nonpreferred color light stimulation also showed differences in terms of the HRV indices compared to the preferred color light stimulation.

Therefore, comparing to the EEG and HRV results in experiment 1 and experiment 2, it shows that the preferred color light stimulation initiated sleep the fastest among all the color light stimulations (including the dark state). It is that this is because the activities of the parasympathetic nervous system were boosted by the preferred color light stimulation, resulting in the fastest inducement of sleep.

The limitations of this study include the subject discomfort due to the use of sensors to measure the EEG, and the fact that the unfamiliarity of the test room could have affected the differences from the normal sleep patterns. In addition, the color light stimulations were consisted of only seven. This is too small to choice the preferred or nonpreferred color. To address this issue, there is a plan to increase the number of color light simulations in the future study. Furthermore, additional studies are planned, with more subjects, so that the measurement of the time of sleep onset using the HRV would show a significant correlation with the cases where the time of sleep onset was measured through the EEG. And, in case of this study, experiments were conducted with subjects who are healthy people. Because this study was a first stage of care of sleep disorder, therefore, there is a plan to conduct experiments with subjects had insomnia.

## 5. Conclusion

In this study, the effects of the colors were used to determine the influence of the color light stimulations on sleep induction. For this purpose, the colors preferred by the individual subjects were surveyed, and the EEG was used to differentiate the awake state from the asleep state. Also, to quantify the physiological effect of the color light stimulation, the ECG was used as well. The results are shown below.

- Depending on the color light stimulation presented, the sleep start time differed. The preferred color light stimulation showed the fastest sleep onset, and with this, it was shown that the preferred color light stimulation influenced the sleep inducement.
- When the subjects entered the asleep state from the alert state, a change occurred in the HRV indices. The time domain indices appeared to increase, and the frequency domain index appeared to decrease. This indicates that the parasympathetic nervous system is activated during the asleep state.
- Based on the color light stimulations, the HRV indices were changed. The time domain indices showed the highest levels with the preferred color light stimulation compared to the stimulations with other colors. Of the frequency domain index, the LF/HF ratio was the lowest with the preferred color light stimulation. These indices that the preferred color light stimulation activate the parasympathetic nervous system most potently.
- In conclusion, the level of index was highest with the preferred color light stimulation, and the frequency domain level of index tended to appear low. That is, the parasympathetic nervous system was more activated with the preferred color light stimulations, contributing to a more stabilized state and therefore sleep inducement. This confirms the potential influence of color light stimulation on sleep.

The results of this study showed that color is closely related to sleep induction. Also, it is expected that the results of this study could be used as the fundamental data for an in-depth study on the physiological effect of colors in the future and could be utilized for creating a sleep environment that could be used for future sleep environment development.

## Figures and Tables

**Table 1:** Subjects' characteristics.

	Experiment 1 (*n* = 10)	Experiment 2 (*n* = 10)
Age (yrs)	26.00 ± 1.77	26.30 ± 1.70
Height (cm)	175.19 ± 3.25	175.79 ± 2.65
Weight (kg)	67.60 ± 5.84	67.52 ± 6.68

**Table 2:** Change of HRV according to awake-asleep state in case of experiment 1 (^∗∗^ *p* < 0.01; awake versus asleep).

	Experiment 1 (*n* = 10)		Experiment 2 (*n* = 10)	
	Awake	Asleep	Awake	Asleep
Mean R-R (ms)^∗∗^	862.22 ± 51.47	907.87 ± 53.38	885.73 ± 42.37	931.44 ± 44.28
SDNN (ms)^∗∗^	34.82 ± 7.34	2.85 ± 6.54	0.59 ± 4.42	39.41 ± 4.54
rMSSD (ms)^∗∗^	1.55 ± 8.66	40.91 ± 10.30	28.21 ± 5.78	37.64 ± 8.15
pNN50 (%)^∗∗^	1.96 ± 7.76	9.27 ± 9.61	9.94 ± 6.67	18.46 ± 8.36
LF/HF ratio (%)	1.57 ± 0.58	1.19 ± 0.28	1.63 ± 0.52	1.26 ± 0.29
